# Development and Performance Evaluation of Residue-Reinforced Recycled HDPE and Bio-Based PP Packaging via Blow Extrusion

**DOI:** 10.3390/polym18111307

**Published:** 2026-05-26

**Authors:** Bruna Basto, Bárbara Freitas, Fernando Leite, João Bessa, Gonçalo Oliveira, Ricardo Neto, Raul Fangueiro

**Affiliations:** 1 Fibrenamics-Institute for Innovation in Fibrous and Composite Materials, 4800-058 Guimarães, Portugal; brunabasto@fibrenamics.com (B.B.); barbarafreitas@fibrenamics.com (B.F.); joaobessa@fibrenamics.com (J.B.); rfangueiro@dem.uminho.pt (R.F.); 2 B4Logic, Rua Principal N° 172, Edifício Prisma, 1°D, Ponte de Vagos, 3840-326 Vagos, Portugal; goncalo.oliveira@b4logic.com; 3 MPlastic, Rua Principal N° 172, Edifício Prisma, Sala D, Ponte de Vagos, 3840-326 Vagos, Portugal; ricardo.neto@mplastic.com; 4 Department of Textile Engineering, University of Minho, 4800-058 Guimarães, Portugal

**Keywords:** polyolefin composites, compounding, blow moulding, mineral fillers, slate powder (SP), bivalve shell powder (BSP), photo-oxidative degradation, mechanical reinforcement, barrier properties

## Abstract

This study investigates the development and performance of polyolefin-based packaging materials reinforced with industrial mineral residues, specifically slate powder (SP) and bivalve shell powder (BSP). High-density polyethylene (HDPE) and polypropylene (PP) matrices were compounded with these fillers and processed by extrusion blow moulding to produce final prototypes. Thermal analyses (TGA and DSC) showed that incorporating SP and BSP does not compromise the thermal stability of the polymer matrices and increases stiffness in the filled formulations. Accelerated ageing (QUV, 200 h) revealed distinct photo-oxidative behaviours. PP and PP + BSP (30%) exhibited increased fragility and moderate colour changes, whereas PP + SP (10%) retained flexibility, indicating a partial protective effect of SP. HDPE-based formulations showed higher intrinsic UV resistance, with HDPE + BSP (30%) displaying excellent colour stability. Tensile tests before and after QUV exposure confirmed that fillers increase stiffness with limited influence on tensile strength. Air permeability results indicated that neat PP and HDPE were below the detection limit. At the same time, filled formulations exhibited measurable permeability, suggesting that filler incorporation may influence gas transport through interfacial effects. Overall, the results show that SP and BSP act as reinforcing additives and can modify functional properties such as stiffness and ageing resistance. However, their influence on barrier performance depends on the formulation and permeation mechanism.

## 1. Introduction

The increasing environmental and societal pressures to reduce plastic waste have driven research toward sustainable alternatives to conventional petroleum-based polymers [1]. Packaging, one of the largest consumers of plastics, represents a key sector where eco-friendly materials can contribute to circular economic strategies. A circular economy prioritises resource efficiency, waste minimisation, and material reuse, emphasising the valorisation of industrial and biological residues to create value-added products [2]. In this context, bio-based and recycled polymers, when combined with functional fillers, offer a promising route to producing high-performance packaging materials while reducing environmental impact.

Polyethylene (PE) and PP are widely used in packaging due to their mechanical robustness, chemical resistance, and processability [3,4]. However, their barrier properties and susceptibility to UV degradation limit their application in certain high-performance packaging applications [5]. Recent studies have demonstrated that incorporating mineral fillers, including industrial by-products and naturally occurring residues, can significantly enhance the functional performance of these polymers [6]. Slate particles [7], bivalve shells [8], rice husk ash [9], and other calcium-rich residues have been explored as fillers to improve the mechanical strength, UV protection, and gas-barrier properties of bio-based HDPE and PP composites. These fillers act primarily through physical reinforcement and increased tortuosity, slowing the diffusion of gases and moisture while maintaining polymer processability [10].

Valorisation of waste materials not only improves polymer properties but also aligns with sustainable production models. BSP, a by-product of the seafood industry, is rich in calcium carbonate and can enhance stiffness and thermal stability in polymer composites [11,12]. SP residues, commonly generated from mining and construction, can improve UV blocking and maintain flexibility when incorporated at appropriate loadings [13]. By repurposing these residues, material development simultaneously addresses waste management challenges and supports circular economy principles, converting otherwise discarded materials into functional resources.

Despite extensive laboratory studies on polymer–residue composites, most research has focused on material characterisation at a small scale, leaving practical applications underexplored. Translating these materials into packaging requires evaluating processing behaviour, such as extrusion or blow moulding, and understanding how filler type and content influence final product performance. High-performance packaging must balance barrier properties, mechanical integrity, and UV stability, while remaining processable for industrial-scale manufacturing. Evaluating these factors is crucial for ensuring that bio-based and residue-reinforced polymers are viable alternatives to conventional plastics [14,15].

The present study builds on previous work showing that SP and BSP fillers enhance the barrier, mechanical, and UV-protective properties of HDPE and PP. Here, the focus shifts to practical implementation, producing blow-extruded bottles from these formulations. The study evaluates both the extrusion process and the functional performance of the resulting bottles, including compression resistance, water vapour and air permeability, UV stability with colour analysis, and tensile behaviour before and after QUV exposure. Tests with standard liquids further assess the bottles’ suitability for real-world applications. Several studies have explored the valorisation of industrial and biological residues as fillers in polymer composites, demonstrating their potential to improve material properties while addressing waste management challenges. For example, SP has been investigated as a reinforcing filler in PP composites, showing that incorporating slate residues does not significantly degrade mechanical strength and can yield systems with performance comparable to pure PP while contributing to environmental waste reduction [10,16,17]. Similarly, mollusc and seashell wastes have been widely studied as bio-fillers in PP and PE matrices. Powdered marine shells have been successfully incorporated into PP and PE composites, yielding thermomechanical properties comparable to those of conventional mineral fillers and enhancing stiffness and modulus [8,18,19].

By integrating residue valorisation, bio-based polymer reinforcement, and practical application testing, this study contributes to the development of sustainable packaging solutions for the detergent, petrochemical, and agrochemical sectors, where they are used to store and transport technical and industrial products.

The findings demonstrate the potential of circular economy strategies to convert waste into high-value materials while improving polymer functionality, advancing the practical use of environmentally responsible HDPE and PP composites in packaging.

## 2. Materials and Methods

### 2.1. Materials

Three composite formulations previously developed and characterised by the authors [20] were selected for this study: recycled HDPE reinforced with 30 wt% BSP, bio-based PP reinforced with 10 wt% SP, and bio-based PP reinforced with 30 wt% BSP. HDPE Repsol Reciclex^®^ 50RX5503, containing 50% post-consumer recycled content from industrial packaging, was supplied by Quimidroga, Lisboa, Portugal. Nexeo Plastics, Lisboa, Portugal provided the biobased PP (Bornewables^TM^, Borealis). Bondyram 5108 and Bondyram 1101 were purchased from IMCD, Porto, Portugal. SP was kindly donated by a company from the central region of Portugal. BS were obtained from Ria de Aveiro, Portugal. Both BS and SP were dried at 60 °C for 12 h and then processed in a jaw crusher. Afterwards, the ground BS and SP were sieved to separate particles smaller than 250 μm. The pellets were produced by extrusion using a twin-screw extruder, Tecnocanto TECN-20-LD 44 (Tecnocanto, Leiria, Portugal), with a screw diameter of 22 mm and a screw diameter/length ratio of LD 44. From zone 1 to zone 6, the temperature profiles used were 185, 185, 175, 170, 160, and 150 °C, respectively. The feeding and extrusion speeds were 35 rpm and 75 rpm, respectively. After extrusion, the obtained filaments were cooled in a cold-water bath and milled into pellets that were used in the present work to manufacture bottles by extrusion.

### 2.2. Methods

#### 2.2.1. Prototypes Production

**Figure 1:**
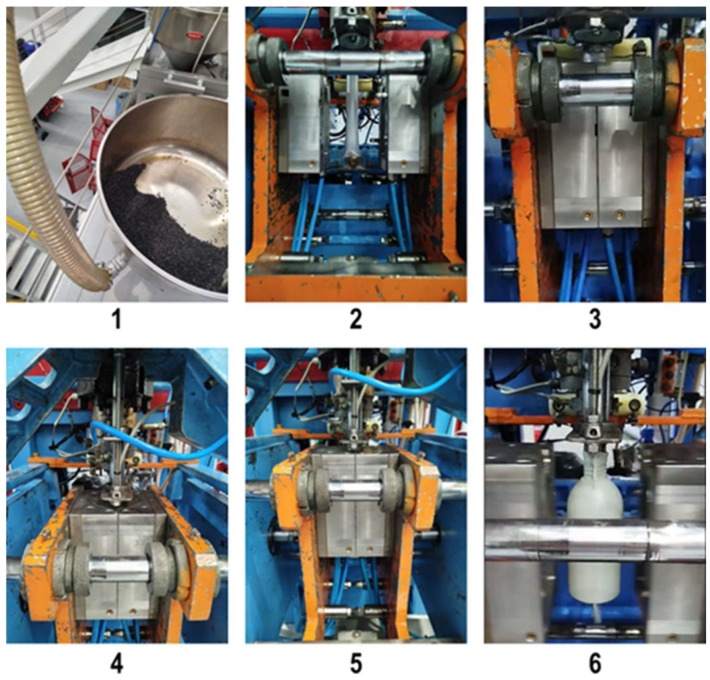
Steps in prototype production: (**1**) feeding, (**2**) pellet extrusion and parison formation, (**3**) parison cutting and mould closing, (**4**) introduction of compressed air into the mould and parison stretching, (**5**) decompression and cooling of the material in the mould, and (**6**) mould opening and final packaging removal.

Bottle prototypes with dimensions of 7 cm in diameter and 18 cm in height, featuring a neck of 2.5 cm in diameter and 2 cm in height, were manufactured by extrusion-blow moulding under industrial conditions. The process involved melting the composite pellets, forming a parison, and shaping it within a closed mould using compressed air. The main stages of the production process are illustrated in Figure 1.

As shown in Figure 1, the procedure comprised the following steps: (1) feeding of the pellets into the extruder; (2) melting and extrusion of the material with parison formation; (3) parison cutting and mould closing; (4) introduction of compressed air into the mould, promoting parison expansion and stretching against the mould walls; (5) decompression and cooling of the material within the mould; and (6) mould opening and removal of the final packaging prototype.

The blow extrusion process was carried out under controlled conditions to ensure uniformity and reproducibility of the prototypes. Key processing parameters, including extrusion temperatures, cycle time, air and water flow rates, and cooling conditions, are summarised in Table 1. These settings were optimised to accommodate the different composite formulations and to maintain consistent parison formation, mould filling, and final product quality.

#### 2.2.2. Thermal Analysis (TGA and DSC)

The thermal behaviour of the formulations was analysed using DSC and TGA, following ISO 11357-1 [21] and ISO 11358 [22], respectively. DSC measurements were performed to determine the melting (T_m_) and crystallisation (T_c_) temperatures and enthalpies (ΔH), and to estimate the degree of crystallinity of the samples. TGA was employed to assess thermal stability and to quantify the inorganic residue arising from the incorporation of mineral fillers. All analyses were conducted on samples collected directly from the prototypes under controlled experimental conditions, ensuring reproducibility and comparability of results across formulations.

#### 2.2.3. Air and Water Permeability

The air permeability was measured using an air permeability tester from TEXTEST instruments (Zurich, Switzerland, model FX 3300) with a differential pressure of 100 Pa and a test area of 20 cm^2^, adapted from Pais et al. [23]. For each sample, at least 10 measurements were taken, and the mean and standard deviation, expressed in L/m^2^.s, were calculated.

The water vapour transmission rate (WVTR) was determined using a water vapour permeability tester in accordance with ASTM E96/E96M-14 [24]. Measurements were conducted over 24 h in a controlled environment (temperature 24 °C, relative humidity 50%) after one hour of stabilisation. Samples for WVTR measurements were cut from the cylindrical walls of the bottles, as this was the only region that allowed the preparation of circular specimens with the required diameter for the testing apparatus. The average sample thickness was approximately 0.6 mm.

Three repetitions were performed per sample, each on a different day to assess reproducibility. The mean and standard deviation were calculated, and the WVTR was determined using the following equation:

$$WVTR\;\left(\frac{\frac{g}{m^{2}}}{day}\right)=\frac{24\times m}{A\times t}$$

where *m* is the mass loss (g), *A* is the exposed sample area (approximately 0.00636 m^2^), and *t* is the time between weighings (t = 24 h).

#### 2.2.4. Mechanical Testing (Compression and Tensile)

**Figure 2:**
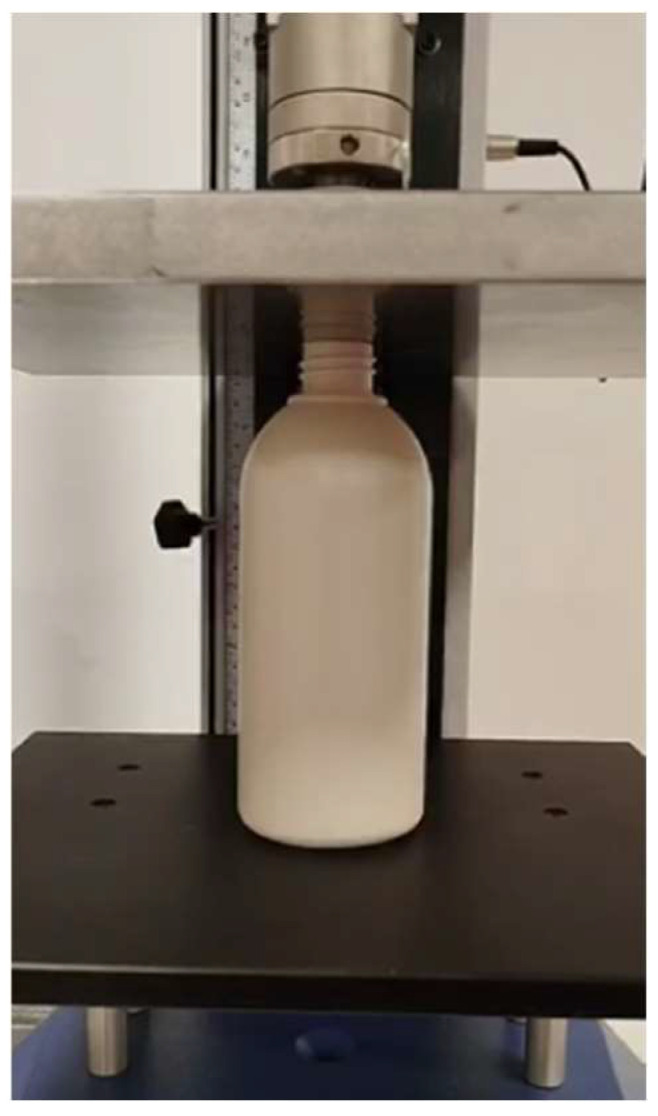
Demonstration of the compression test performed on intact prototypes.

Mechanical tests were performed to evaluate the behaviour of the formulations under static loading. Tensile tests were conducted in accordance with ISO 527 [25] using a universal testing machine at a crosshead speed of 5 mm/min, with a 50 kN load cell and a grip distance as specified in the standard. Compression tests were performed following ISO 604 [26]. For tensile testing, specimens were cut directly from the prototypes in the 1BA geometry specified by the standard, whereas compression tests were carried out using the intact prototypes (Figure 2).

#### 2.2.5. Accelerated Ageing (QUV)

Accelerated ageing of the formulations was performed in a QUV chamber according to ISO 4892-3 [27]. Samples were exposed to controlled cycles of UV radiation (irradiance of 0.76 W·m^−2^·nm^−1^; 8 h of light at 60 °C) and moisture condensation (4 h at 50 °C) using UVA-340 lamps for a total duration of 200 h.

After ageing, colour changes in the samples were quantified using spectrophotometry and the CIELAB colour system to ensure an objective and comparable assessment of chromatic stability. The measured colour coordinates included lightness (L*), red–green axis (a*), and blue–yellow axis (b*). From these values, the total colour difference (ΔE) was calculated to establish acceptance criteria. A ΔE ≤ 3 indicates an acceptable colour change, whereas ΔE > 5 denotes a significant colour variation, which may be considered a rejection criterion.

#### 2.2.6. Statistical Analysis

Statistical analyses were performed using GraphPad Prism version 10.6.1 (GraphPad Software, San Diego, CA, USA) to compare the new formulations with their respective virgin polymers. Data are presented as mean ± standard deviation (SD). Statistical significance was set at *p* < 0.05.

## 3. Results and Discussion

### 3.1. Prototypes Production

**Figure 3:**
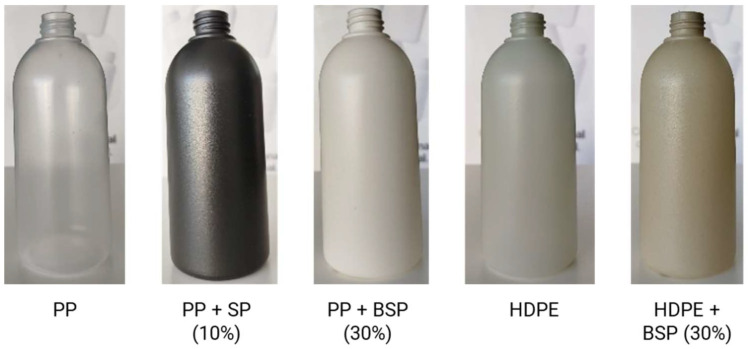
Prototypes successfully obtained via blow extrusion.

Bottle prototypes were produced using the selected composite formulations (Figure 3).

Figure 3 illustrates the final prototypes, showcasing their uniform shape, surface finish, and structural integrity. These results confirm the suitability of the developed pellets for blow extrusion and demonstrate the potential of the bio-based and residue-reinforced composites for sustainable packaging applications.

The literature confirms that both slate and shell residues can serve as sustainable fillers in polyolefin composites. However, most published research has focused on compounding and characterising pellets or compression-moulded specimens. As far as we know, no studies have reported the production of actual packaging containers (e.g., blow-moulded bottles) using these residue-reinforced composites. The present study addresses this gap by applying residue-filled HDPE and PP formulations to the blow extrusion process, producing fully formed prototypes and evaluating their performance across multiple functional criteria.

### 3.2. Thermal Analysis (TGA and DSC)

**Figure 4:**
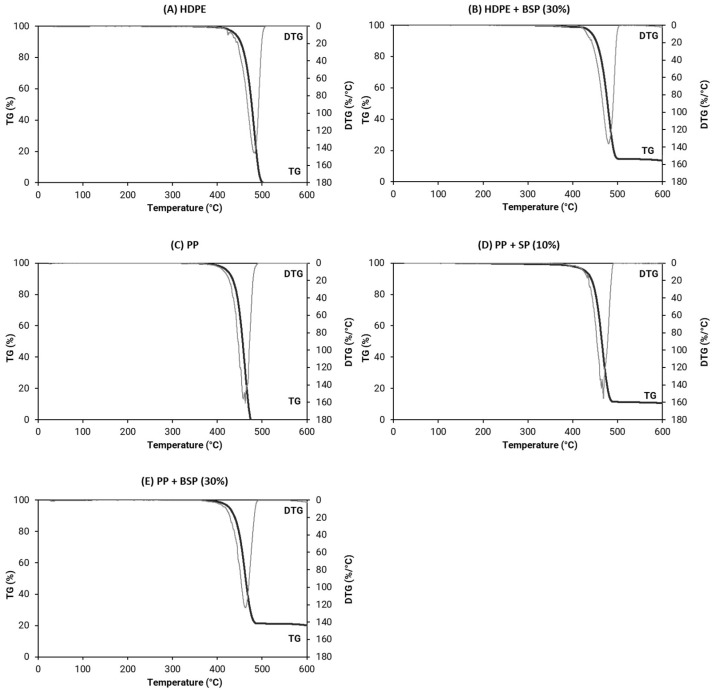
TGA curves of the studied samples: (**A**) HDPE, (**B**) HDPE + BSP (30%), (**C**) PP, (**D**) PP + SP (10%), and (**E**) PP + BSP (30%).

TGA was used to evaluate the thermal stability of the composite formulations, and the results are presented in Figure 4. The figure illustrates the weight-loss profiles of the different samples as a function of temperature, highlighting the influence of the incorporated slate and bivalve shell residues on the thermal degradation behaviour. These data provide insights into the composites’ thermal resistance and the proportion of inorganic content derived from the fillers.

Figure 4 shows that for both polymer matrices, degradation onset and completion temperatures were consistent with the expected thermal stability of neat PP and HDPE, indicating that the addition of SP and BSP did not compromise polymer integrity. In PP-based formulations, thermal degradation began at approximately 390 °C and was essentially complete around 490 °C. The PP + SP (10%) formulation exhibited a final residue of approximately 10%, closely matching the nominal filler content, confirming the predominantly inorganic nature of SP and its effective incorporation into the polymer matrix. In contrast, the PP + BSP (30%) formulation showed a lower final residue (~20%), despite the nominal 30% filler loading. This discrepancy likely reflects the heterogeneous composition of BSP, which may contain thermally labile organic fractions or partially degrade during extrusion, reducing the detectable inorganic fraction. Similar trends were observed in HDPE-based formulations, with degradation onset around 400 °C and completion near 500 °C. HDPE + BSP (30%) again exhibited a lower residual mass than the nominal filler content.

These results are consistent with previously reported findings in the literature, which indicate that HDPE and PP composites reinforced with mollusc or BSP powders generally preserve the inherent thermal stability of the polymer matrix. The final residual mass is often observed to be lower than the nominal filler content, a behaviour typically attributed to the presence of thermally labile organic constituents within the BSP or to partial degradation occurring during melt processing. Such outcomes highlight the capacity of these biological residues to serve as reinforcing fillers without significantly compromising the polymer’s thermal integrity, while also reflecting the heterogeneous nature of the BSP material [28,29].

Similarly, studies on SP-reinforced polymers demonstrate that incorporating SP, whether as fibres or powder, preserves the polymer’s thermal behaviour and provides a stable inorganic fraction. Carbonell-Verdú et al. [10] reported that HDPE reinforced with slate fibres retained its thermal stability during injection moulding, while Khan et al. [17] found that waste SP incorporated into PLA composites maintained polymer thermal behaviour and contributed a thermally stable residue.

Similarly, studies on SP-reinforced polymers have demonstrated that the incorporation of slate fibres or waste slate powder preserves polymer thermal behaviour and contributes a thermally stable inorganic fraction [10,17], supporting the feasibility of using industrial mineral residues without compromising polymer performance.

Overall, the TGA results confirm that the developed formulations are thermally robust and that the nature of the fillers, highly stable inorganic SP versus partially degradable BSP, directly influences the residual mass. These results are particularly relevant for industrial processing, demonstrating that the formulations can withstand thermal loads typical of extrusion-based manufacturing and provide a foundation for further studies on the processing and functional performance of residue-reinforced polyolefin packaging.

DSC was employed to investigate the thermal transitions and crystallinity of the developed HDPE- and PP-based formulations. This analysis provides critical insights into the melting and crystallisation behaviour of the polymers, as well as the potential effects of the incorporated mineral fillers (SP and BSP) on the polymer microstructure. By evaluating these parameters, it is possible to assess how the residues influence polymer chain mobility, crystal formation, and overall thermal performance, which are directly related to the mechanical properties and processability of the composites. In the present study, melting (ΔHm), enthalpies, and degree of crystallinity (Xc) were determined for all formulations. The crystallinity of the formulations containing mineral residues (SP and BSP) was calculated using the following equation:

$$Xc\;(\%)=\frac{\Delta Hm\;(J/g)}{{\Delta Hm}^{100\%}(J/g)}\times 100\%$$

where Xc represents the degree of crystallinity, ΔHm is the measured melting enthalpy of the sample, and ΔHm^100%^ corresponds to the melting enthalpy of the pure polymer. The ΔHm^100%^ values used were taken from the literature for fully crystalline polymers: 207 J/g for PP [30,31] and 293 J/g for HDPE [32]. The results obtained are summarised in Table 2.

The results show that, for PP formulations, neat PP exhibited a Tm of 142.5 °C. The addition of 10% SP resulted in a slight increase in Tm to 144.3 °C, which may indicate a modest promotion of crystalline order due to heterogeneous nucleation effects. Conversely, the PP + BSP (30%) formulation showed a moderate reduction in Tm to 140.1 °C, suggesting that the nature of BSP, combined with its higher loading, disrupts perfect crystalline packing to a greater degree. The normalised ΔHm values decreased in both residue-filled samples relative to neat PP, as expected given the presence of inorganic filler phases that do not contribute to polymer melting. Xc shows contrasting effects, as PP + SP (10%) exhibits relatively higher crystallinity, highlighting the effective nucleating role of SP. In contrast, PP + BSP (30%) exhibits lower crystallinity, likely due to restricted polymer chain mobility from the high filler content and the heterogeneous nature of BSP.

In HDPE-based composites, neat HDPE exhibited a Tm of 135.1 °C, while the HDPE + BSP (30%) formulation showed a slight reduction to 130.4 °C. This behaviour is consistent with reports in the literature that fillers can disturb the uniformity of crystal lamellae and slightly depress Tm without radically altering the melting mechanism [33]. The ΔHm of neat HDPE likewise decreased upon incorporation of BSP, consistent with the higher inorganic content. Nevertheless, the calculated Xc for HDPE + BSP (30%) remained relatively high, indicating that, despite the presence of a significant load of mineral residue, the overall semicrystalline morphology of HDPE is largely preserved. This reflects the intrinsic tendency of HDPE to form extensive crystalline domains due to its linear chain structure and low branching, a behaviour widely reported in DSC studies where HDPE displays higher crystallinity relative to PP when normalised against literature values of ΔH^100%^ [34].

These trends align with broader observations in the polymer composite literature. The ability of particulate fillers to act as heterogeneous nucleating agents and to influence the degree of crystallinity has been documented across multiple polymer systems, with modest adjustments in crystallinity attributed to interfacial interactions and filler surface effects. Although the magnitude of these effects depends on filler type, morphology, and concentration, the retention of significant crystallinity in residue-filled samples supports the conclusion that SP and BSP can be incorporated without severely compromising the primary semicrystalline structure of PP and HDPE [35,36].

Overall, the DSC results indicate that SP, particularly at lower loadings, may enhance crystallinity by providing nucleation sites, whereas higher BSP loadings restrict polymer chain organisation, resulting in lower crystallinity.

### 3.3. Air and Water Permeability

**Figure 5:**
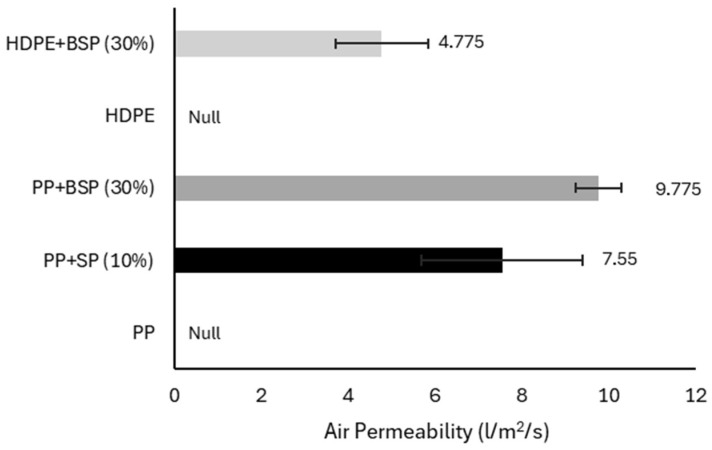
Air permeability of the studied formulations (PP, PP + BSP (10%), PP + BSP (30%), HDPE and HDPE + BSP (30%)) was measured at a differential pressure of 100 Pa and a test area of 20 cm^2^. Values are the mean ± SD (n = 3).

**Figure 6:**
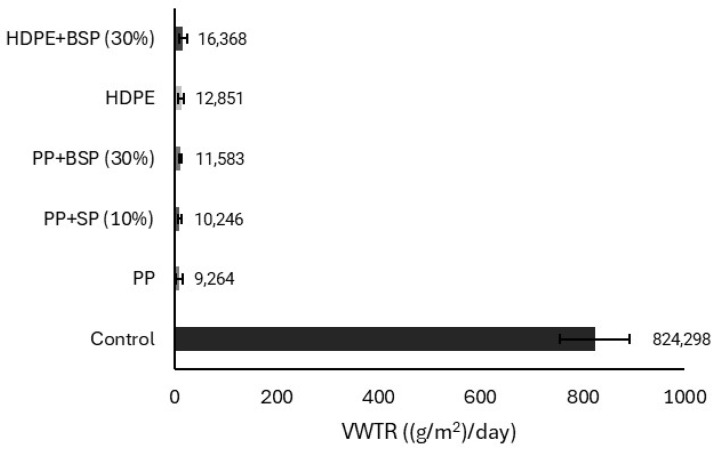
WVTR of the studied formulations (PP, PP + BSP (10%), PP + BSP (30%), HDPE and HDPE + BSP (30%)) under controlled conditions (24 °C and 50% relative humidity). Values are the mean ± SD (n = 3).

The barrier performance of the developed formulations was evaluated using air permeability and WVTR measurements, key parameters for assessing the suitability of materials for packaging applications. The results for the different formulations are presented in Figure 5 and Figure 6, which show the influence of incorporating SP and BSP on air and moisture transport through the polymer matrices.

In the air permeability tests, neat PP and HDPE samples showed zero permeability under the experimental conditions. It is important to emphasise that these results do not necessarily indicate absolute impermeability; rather, the measured air flux through these materials was below the testing apparatus’s detection limit. This limitation is common in air permeability testing when dealing with dense polymeric materials and small pressure gradients.

For the residue-filled formulations, measurable air permeability values were observed, indicating that incorporating mineral fillers affects gas transport pathways. PP + SP (10%) and PP + BSP (30%) exhibited average permeability values of 7.55 and 9.78 L/m^2^.s, respectively, whereas HDPE + BSP (30%) had a mean permeability of 4.78 L/m^2^.s. The increase in air permeability upon filler addition is consistent with the fact that the incorporation of particulate fillers can introduce interfacial regions, microvoids, or preferential pathways that facilitate air diffusion, especially at higher filler loadings or when filler dispersion is imperfect. Such mechanisms have been widely discussed in the context of polymer composites, where enhanced tortuosity may improve the barrier to some permeants but also lead to localised heterogeneities that facilitate gas transport routes when filler-matrix adhesion is suboptimal [37,38].

Overall, the air permeability results demonstrate that the inclusion of SP and BSP influences gas transport behaviour, likely by modifying the microstructure and creating diffusion pathways. However, the observed permeability values remain within the range typical for semi-rigid polymer films used in packaging and do not indicate a significant loss of barrier functionality. These findings are consistent with the literature on polymer composites, in which barrier properties can be tailored by filler type and content. Still, absolute impermeability is rarely achieved in single-layer systems without specialised barrier layers.

In the WVTR results, the standard sample exhibited a very high permeability (824.30 (g/m^2^)/day). In contrast, all polymer-based formulations showed substantially lower values, and no statistically significant differences were observed between the polyolefin-based polymers.

Although no statistically significant differences were observed, the literature indicates that minor variations can arise depending on the filler type, morphology, and dispersion, as well as the intrinsic barrier properties of the base polymer. For example, studies on polymer nanocomposites have shown that increasing filler content can enhance barrier properties by reducing the effective free volume and disrupting direct diffusion pathways, thereby lowering WVTR values as predicted by classical tortuosity models [39]. Similarly, in filled PLA systems, increasing crystallinity, promoted by adding certain fillers, was associated with decreased WVTR, as the more ordered structure reduces the diffusivity and solubility of WP in the polymer matrix [40].

The absence of pronounced differences between the neat and residue-filled polyolefin formulations in terms of WVTR in the present work may be attributed to the inherently low permeability of both PP and HDPE, which limits the sensitivity of the measurement to filler effects at the tested loadings. Such findings emphasise that barrier improvements are most significant when the filler geometry and dispersion are optimised to maximise the tortuous path effect.

These findings highlight the potential of mineral residues as functional fillers in polymer composites for packaging applications, where barrier performance is a critical parameter that should not be compromised.

### 3.4. QUV

The visual appearance of the developed formulations before and after accelerated ageing (QUV, 200 h) was evaluated to qualitatively assess the effects of UV radiation, temperature, and humidity on the samples’ surface integrity and colour stability (Table 3).

For PP-based formulations, the observed increase in brittleness in both neat PP and PP + BSP (30%) indicates photo-oxidative degradation of the polymer matrix. PP is particularly susceptible to UV-induced chain scission due to the presence of tertiary carbon atoms in its backbone, which are susceptible to radical formation under UV exposure [41]. Bendjaouahdou and Bensaad [42] investigated the effects of thermal and ultraviolet ageing on natural rubber-PP blends using optical microscopy. The resulting images, in agreement with those obtained in the present work, revealed the formation of surface cracks and additional debonded rubber domains from the PP matrix, induced by both UV and thermal ageing.

In contrast, the PP + SP (10%) formulation maintained a relatively ductile behaviour after ageing, suggesting a stabilising effect of SP. In a previous study, it was demonstrated that the incorporation of SP markedly reduces UV permeability, with transmittance approaching 0% at a filler content of only 5% [20]. Thus, these results can be attributed to SP’s ability to act as a physical barrier, limiting UV penetration and thereby slowing the photo-oxidative degradation of the polymer matrix. Additionally, the inherent colour and morphology of SP may contribute to UV shielding, further enhancing stability.

For HDPE-based formulations, including HDPE + BSP (30%), no significant changes in flexibility or brittleness were observed after QUV exposure. This behaviour highlights the inherently higher resistance of HDPE to UV degradation compared to PP. The chemical structure of HDPE makes it less susceptible to chain scission, contributing to better retention of mechanical properties under photo-oxidative conditions [43].

Based on the results, the exclusion of the PP and PP + BSP (30%) formulations from further analysis is justified, as their structural integrity was severely compromised under the applied ageing conditions. This underscores the critical role of both matrix selection and filler type in designing materials for UV-exposed applications.

To complement the analysis, the colour changes of the samples were monitored using spectrophotometry in the CIELAB colour space (Table 4), allowing quantification of the chromatic variations induced by ageing and evaluation of the protective effect of the mineral fillers incorporated into the matrix.

The PP + SP (10%) formulation exhibited a moderate colour change (ΔE = 3.73), indicating partial protection against UV-induced discolouration. This is consistent with the barrier role of SP, which can reduce the extent of photo-oxidative reactions on the surface.

Neat HDPE showed the highest ΔE value (4.14), indicating greater susceptibility to surface degradation. On the other hand, HDPE + BSP (30%) exhibited a very low ΔE value (0.66), demonstrating excellent colour stability. This result indicates that BSP also plays a protective role in the HDPE formulation, whereas the same effect was not observed in the PP + BSP (30%) formulation. This result shows a synergistic effect between the HDPE matrix and the BSP reinforcement in reducing radiation penetration into the material.

Basto et al. [20] also demonstrates BSP’s protective effect on UV transmittance at the same concentration used in the present study. When 30% of BSP is incorporated, the value of transmittance drops to 0%.

Overall, the results demonstrate that incorporating mineral residues significantly affects the photo-oxidative behaviour of polyolefin-based materials. While PP is inherently more susceptible to UV degradation, the addition of SP can partially mitigate this effect. In HDPE systems, both the matrix and the incorporation of BSP contribute to enhanced stability, particularly in terms of colour retention. These findings confirm the potential of mineral fillers not only as reinforcing agents but also as functional additives that improve durability under environmental exposure.

### 3.5. Mechanical Testing (Compression and Tensile)

**Figure 7:**
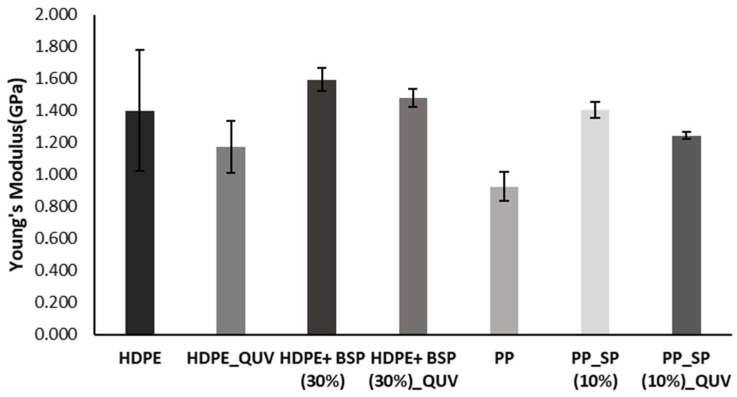
Young’s modulus (GPa) of polyolefin-based materials (HDPE, HDPE + BSP (30%), PP and PP + SP (10%)) before and after QUV exposure.

**Figure 8:**
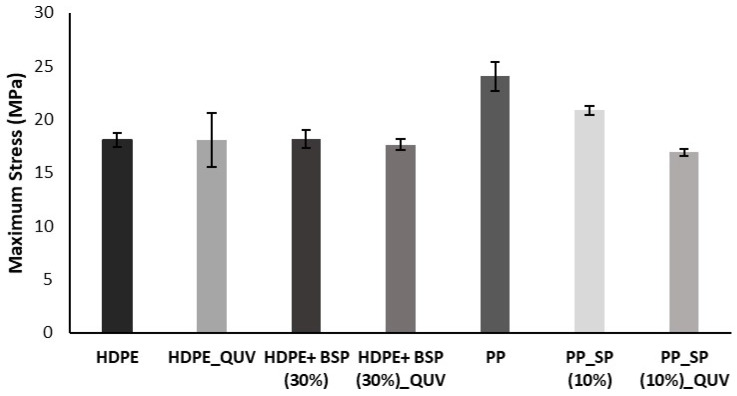
Maximum stress (MPa) of polyolefin-based materials (HDPE, HDPE + BSP (30%), PP and PP + SP (10%)) before and after QUV exposure.

The tensile behaviour of the formulations that demonstrate promising results in the QUV test was evaluated to assess the influence of mineral fillers (SP and BSP) and QUV on stiffness and strength under static loading. The results for Young’s modulus and maximum stress are presented in Figure 7 and Figure 8.

The addition of mineral fillers commonly affects the stiffness and strength of polyolefin composites, with the specific outcome depending on filler type, content, dispersion, and polymer-matrix interaction. In many studies, rigid inorganic fillers lead to an increase in Young’s modulus due to restriction of polymer chain mobility and load transfer from the matrix to the filler phase. For example, similar trends have been documented in PP and PE composites reinforced with mineral or hybrid fillers, where increasing filler content resulted in enhanced stiffness but also potential reductions in ductility [44].

The experimental Young’s modulus values for neat HDPE were lower than those observed in the residue-filled formulations, confirming the reinforcing effect of the incorporated mineral fillers. Both HDPE + BSP (30%) and PP + SP (10%) exhibited higher stiffness compared to their respective unfilled matrices, indicating effective load transfer between the polymer matrix and the rigid filler particles. When analysing the effect of QUV, distinct behaviours were observed across formulations. For neat HDPE, no statistically significant differences were found between unaged and aged samples, suggesting that the material maintains its stiffness under the applied photo-oxidative conditions. This stability has been reported previously, with HDPE showing relatively good resistance to short-term UV exposure due to its semi-crystalline structure and limited oxygen diffusion [45].

In contrast, the HDPE + BSP (30%) formulation showed a statistically significant difference (* *p* < 0.05) after QUV exposure. However, no significant differences were observed when compared with virgin HDPE. The tensile results for HDPE exhibited greater variability, reflected in a higher standard deviation, whereas HDPE + BSP (30%) showed a lower standard deviation, indicating more homogeneous behaviour among the tested samples. The small differences observed in HDPE + BSP (30%) could be attributed to the heterogeneous nature of BSP, which may contain residual organic components or interfacial regions that promote localised degradation. Such effects can alter the polymer–filler interface, thereby affecting stiffness.

A more pronounced effect was observed for PP + SP (10%), where a highly significant difference (** *p* < 0.01) was found after QUV exposure. This suggests that this formulation is more sensitive to photo-oxidative degradation. PP, as was already mentioned, is known to be more susceptible to UV-induced chain scission compared to PE, and the mineral fillers can further influence this behaviour by acting as sites for stress concentration or by modifying the oxidation kinetics [20,42]. Additionally, while SP can block some UV light and may increase stiffness, its interactions with the polymer matrix and potential dispersion heterogeneities could compromise long-term stability under UV exposure.

Regarding the maximum tensile stress, HDPE values are close to 18 MPa before and after ageing, with no significant differences observed. The same was observed in HDPE + BSP (30%) and PP + SP (10%) formulations, indicating that, despite a reduction in modulus, the tensile strength is not significantly affected by QUV exposure, and the mineral filler does not compromise the material’s mechanical strength.

Overall, the tensile test results demonstrate that incorporating mineral fillers, both BSP and SP, increases stiffness and mechanical stability after UV ageing, without significantly compromising the maximum tensile stress. These results reinforce the potential of the developed formulations for rigid packaging applications exposed to environmental conditions.

Previous FTIR analyses reported in an earlier publication [20] showed that the characteristic absorption bands of HDPE and PP were preserved after incorporating SP and BSP, indicating predominantly physical interactions between the fillers and the polymer matrices. The same study also demonstrated the strong UV-shielding capability of SP, with transmittance values approaching 0% at low filler contents. These findings are consistent with the QUV ageing results obtained in the present work, where PP and PP + BSP (30%) became brittle after exposure, while PP + SP (10%) maintained its flexibility, suggesting that the UV-blocking effect of SP contributes to reducing the photo-oxidative degradation of the PP matrix.

## 4. Conclusions

The present work demonstrates the potential of industrial mineral residues as reinforcing fillers in polyolefin-based packaging materials. TGA and DSC analyses confirmed that both SP and BSP preserve the thermal stability of HDPE and PP matrices while increasing stiffness. QUV ageing results showed formulation-dependent photo-oxidative behaviour, with SP providing partial protection in PP-based systems and HDPE exhibiting higher intrinsic UV resistance. Tensile testing indicated increased stiffness with limited impact on tensile strength after ageing.

However, air permeability results revealed that neat PP and HDPE were below the detection limit. In contrast, filler addition yielded measurable permeability, suggesting that filler incorporation may introduce interfacial pathways that affect gas transport.

Overall, SP and BSP act as reinforcing additives with beneficial effects on mechanical performance and ageing behaviour. Still, their influence on barrier properties is not universally positive and depends on the polymer–filler system and permeant considered. These findings highlight the importance of balancing reinforcement and barrier performance in the design of filled polyolefin packaging systems.

## Figures and Tables

**Table 1:** Production conditions for the prototypes by blow extrusion.

Processing Parameter	Value
Extrusion temperature (°C)	Zone 1: ≈180; Zone 2: ≈180; Zone 3: ≈175; Zone 4: ≈175
Cycle time (s)	≈17–20 (180 to 212 bottles per hour)
Cooling time	≈10
Air flow rate (m^3^/h)	≈35
Air pressure (bar)	≈7.40
Die-down delay (s)	0.5
Water flow rate (m^3^/h)	≈80
Water inlet pressure (bar)	4.3 ± 0.5
Water outlet pressure (bar)	3.8 ± 0.5
Water inlet temperature (°C)	7.8 ± 1
Water outlet temperature (°C)	10.8 ± 1

**Table 2:** Values obtained from DSC analysis: melting temperature (Tm), melting enthalpy (ΔHm), and degree of crystallinity (Xc).

Formulation	Tm (°C)	ΔHm (J/g)	Xc (%)
PP	142.5	−76.93	37
PP + SP (10%)	144.3	−62.77	30
PP + BSP (30%)	140.1	−76.93	24
HDPE	135.1	−172.3	59
HDPE + BSP (30%)	130.4	−127.8	44

**Table 3:** Visual appearance of the studied formulations before and after accelerated ageing in a QUV chamber (200 h exposure under UVA-340 radiation).

Formulation	Before QUV	After 200 h QUV
PP	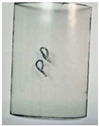	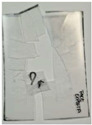
PP + SP (10%)	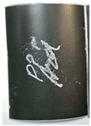	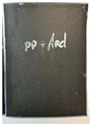
PP + BSP (30%)	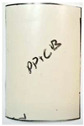	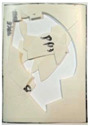
HDPE	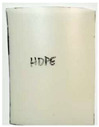	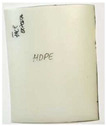
HDPE + BSP (30%)	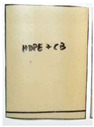	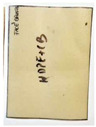

**Table 4:** Analysis of colour change before and after the QUV test.

Formulation	Before QUV	After QUV	ΔE
PP + SP (10%)	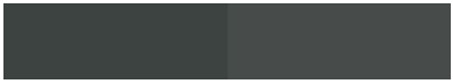		3.73
HDPE	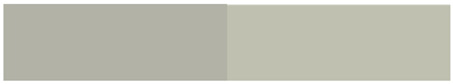		4.14
HDPE + BSP (30%)	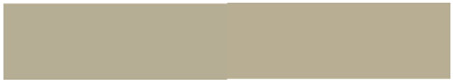		0.66

## Data Availability

The original contributions presented in this study are included in the article. Further inquiries can be directed to the corresponding author.

## Abbreviations

The following abbreviations are used in this manuscript:

BSP	Bivalve shell powder
SP	Slate powder
HDPE	High-density polyethylene
PP	Polypropylene
TGA	Thermogravimetric analysis
DSC	Differential scanning calorimetry
UV	Ultraviolet
WVTR	Water vapour transmission rate
